# Supplementary material for: Electric-field driven nuclear dynamics of liquids and solids from a multi-valued machine-learned dipolar model
Source: NPJ Comput Mater. 2025 Oct 13;11(1):304. doi: 10.1038/s41524-025-01751-x (PMC12518137; doi:10.1038/s41524-025-01751-x)
Supplement: Supplementary file 1 — Supplementary Information [file 41524_2025_1751_MOESM1_ESM.pdf]

Electric-Field Driven Nuclear Dynamics of Liquids and Solids from a Multi-Valued Machine-Learned Dipolar Model
Supplementary Information

Elia Stocco,[1] Christian Carbogno,[2] and Mariana Rossi[1]

[1] *MPI for the Structure and Dynamics of Matter, Hamburg, Germany*
[2] *Theory Department, Fritz Haber Institute of the MPS, Faradayweg 4-6, 14195 Berlin, Germany*

SUPPLEMENTARY SECTION 1. FAILURE OF A SINGLE-VALUED MACHINE-LEARNING MODEL

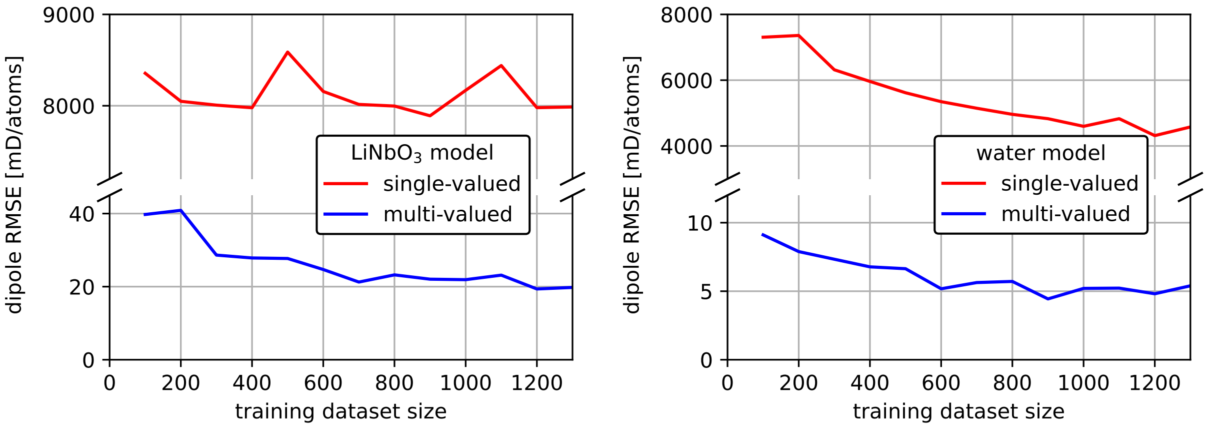

Supplementary Figure 1. Training of dipole ML models for polar systems including strongly out-of-equilibrium configurations. Specifically, the atomic structures used to evaluate the oxidation numbers (see example in Fig. S2 for water) have been added to both the train and test datasets, for $LiNbO_3$ (left) and water (right). The loss function (RMSE in milli-Debye) shows a clear failure of the single-valued model in both cases. We note that for water, removing the "dissociated" configurations shown in Fig. S2 would yield successful training for a single-valued model because the combined oxidation number $\mathcal{N}_I$ of the diffusing molecules is zero.

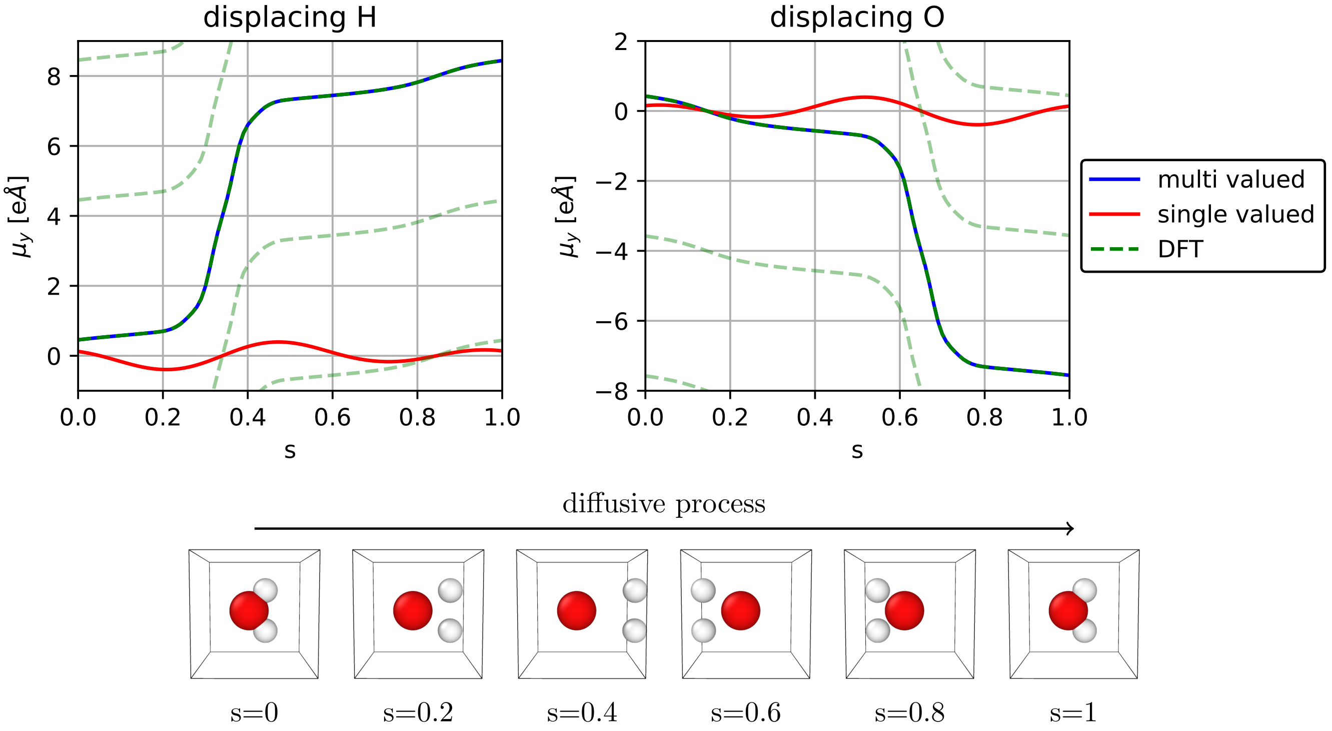

Supplementary Figure 2. Predicted dipole values (along the y-axis) of both the single- and multi-valued model of water along the path of Fig.1b and c of the main text. The models used are the ones whose learning curves are shown in Fig. S1. The left panel shows the trajectory where the oxygen atom is kept fixed while the hydrogen atoms diffuse to a neighboring periodic replica (also shown in the bottom plot), vice versa in the right panel. The DFT values are reported (in green) for different branches as well. The multi-valued model is always in excellent agreement with the DFT data. The single-valued model roughly agrees with the data for $s \approx 0$ (mod 1), where the coordinate s is defined as in the main text, while for larger values of s it completely diverges from the data by even missing the qualitative behavior and slope.

SUPPLEMENTARY SECTION 2. FREQUENCY-DEPENDENT VIBRATIONAL DIELECTRIC PROPERTIES

Supplementary Note 1

The following formula has been used to compute the infrared absorption spectrum $\alpha(\omega)n(\omega)$ of bulk water using the dipole time series [1]:

$$\alpha\left(\omega\right)n\left(\omega\right)=\frac{\pi\omega}{3\hbar c\Omega\varepsilon_0}\left(1-e^{-\beta\hbar\omega}\right)C_{\boldsymbol{\mu}\boldsymbol{\mu}}\left(\omega\right) \tag{S1}$$

where $\alpha\left(\omega\right)$ is the Beer-Lambert absorption coefficient, $n\left(\omega\right)$ is the refractive index of the material, $\beta=1/k_BT$, $\hbar$ the reduced Planck constant, c is the speed of light, ε_0 is the vacuum permittivity, Ω the system volume, and $C_{\boldsymbol{\mu}\boldsymbol{\mu}}\left(\omega\right)$ is the Fourier transform of the standard time correlation function $C_{\boldsymbol{\mu}\boldsymbol{\mu}}\left(t\right)$ of the dipole $\boldsymbol{\mu}$ with itself. By using the relation between the standard $C\left(\omega\right)$ and the Kubo transformed $\tilde{C}\left(\omega\right)$ time correlation function [2] one can re-express the previous expression as follows:

$$\alpha\left(\omega\right)n\left(\omega\right)=\frac{\pi\beta\omega^2}{3c\Omega\varepsilon_0}\tilde{C}_{\boldsymbol{\mu}\boldsymbol{\mu}}\left(\omega\right) \tag{S2}$$

where $\tilde{C}_{\boldsymbol{\mu}\boldsymbol{\mu}}\left(\omega\right)$ is approximated by using molecular dynamics with the classical time correlation function of the dipole with itself:

$$\tilde{C}_{\boldsymbol{\mu}\boldsymbol{\mu}}\left(\omega\right)\approx\int_{-\infty}^{+\infty}dt\,e^{-i\omega t}\left\langle\boldsymbol{\mu}\left(0\right)\cdot\boldsymbol{\mu}\left(t\right)\right\rangle_\beta \tag{S3}$$

The frequency-dependent dielectric susceptibility $\chi\left(\omega\right)$ is evaluated in a similar manner [1]:

$$\chi\left(\omega\right)=-\frac{\beta}{3\varepsilon_0\Omega}\int_{0}^{+\infty}dt\,e^{-i\omega t}\left\langle\boldsymbol{\mu}\left(0\right)\cdot\dot{\boldsymbol{\mu}}\left(t\right)\right\rangle_\beta \tag{S4}$$

SUPPLEMENTARY SECTION 3. DIPOLE MODEL FROM PERIODIC STRUCTURES

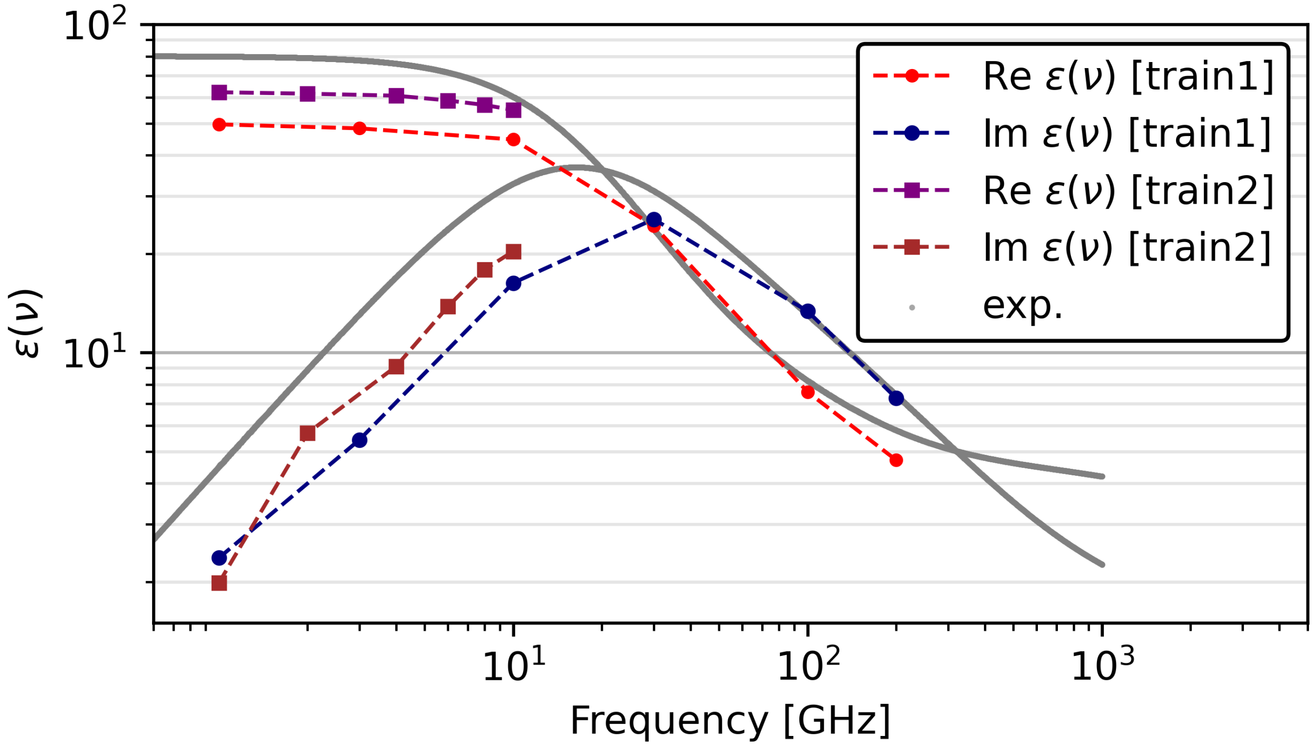

Supplementary Figure 3. The figure presents the real and imaginary parts of the dielectric permittivity of liquid water at 300 K over the 1–135000 GHz frequency range, obtained using the non-equilibrium applied electric-field method (see the main text). The blue and red datasets (circles, train1) correspond to the results discussed in the main text, with the original training set for dipoles obtained from large water clusters. The purple and brown datasets (squares, train2) were obtained from a model trained on periodic water structures, instead of droplets. The dataset comprised of 1000 structures, each containing 32 water molecules at standard density. The dipoles were evaluated using Berry-phase polarization implementation in the FHI-aims code and the revPBE functional. The results indicate that data from periodic structures better captures the bulk regime and predicts a dielectric constant closer to the experimental value. Ramaining differences to experimental results are attributed to the functional used here (revPBE).

SUPPLEMENTARY SECTION 4. QUANTUM INFRARED SPECTRUM OF LIQUID WATER

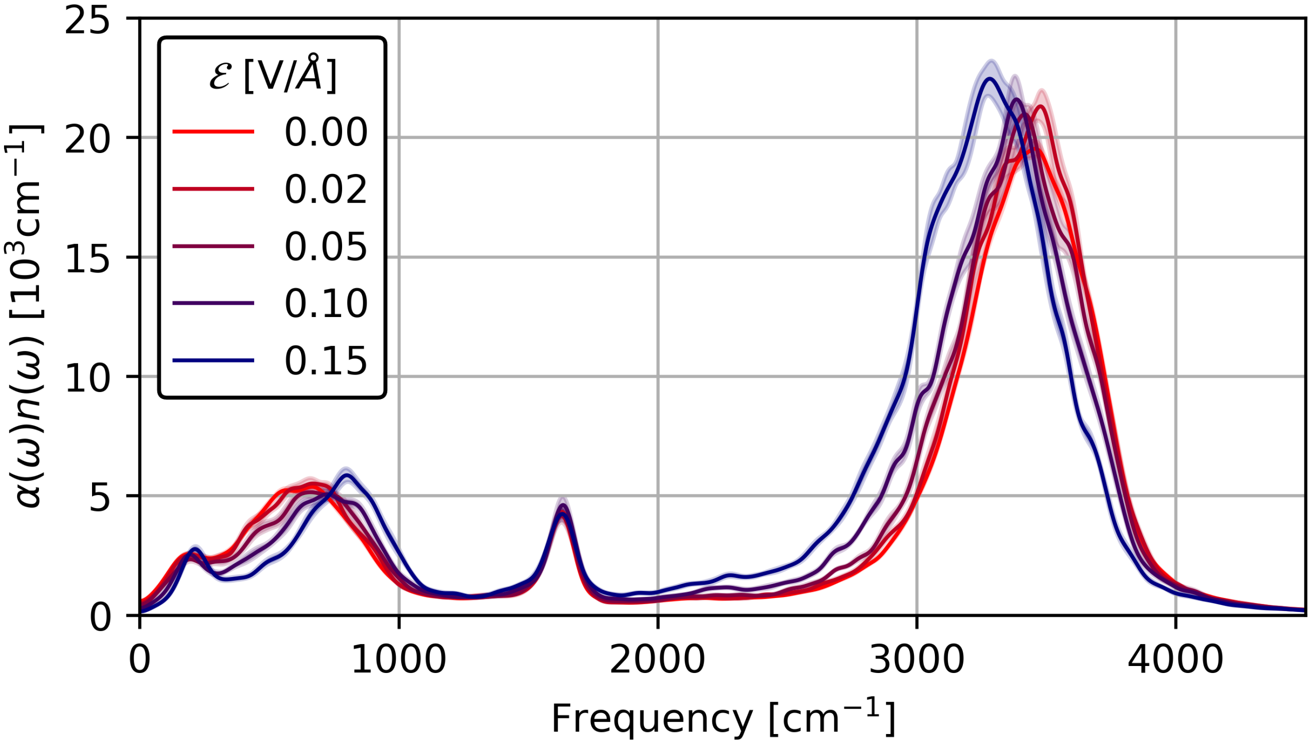

Supplementary Figure 4. The infrared absorption spectrum of liquid water at varying electric field as obtained simulated with thermostatted ring-polymer molecular dynamics (TRPMD) [3], which includes nuclear quantum statistics. These spectra were obtained in a similar manner as done for the classical case shown in the main text. The values of the dipoles were obtained as the mean over the 32 beads. For each field intensity, 8 thermalized simulations of 10ps each were run with a time-step of 0.25 fs at 300 K using a global path integral Langevin equation thermostat (`pile_g`) with $\tau = 500$ fs and $\lambda = 0.5$ [4].

SUPPLEMENTARY SECTION 5. PHASE TRANSITION OF $LINBO_3$

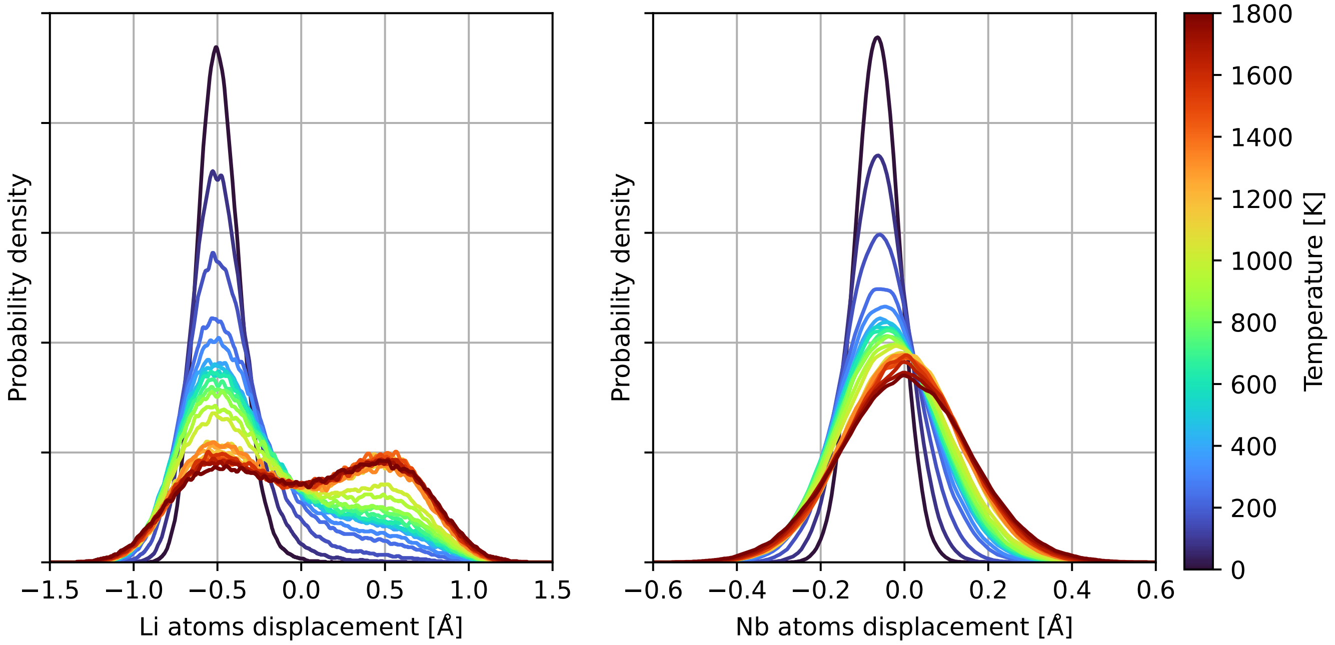

Supplementary Figure 5. Normalized histograms of atomic displacements for Li and Nb atoms at various temperatures with respect to the paraelectric structure. The displacement distributions have been normalized by the maximum value and then smoothed using a Gaussian filter. These results are in agreement with what reported in Ref. [5], i.e. a bimodal distribution above the Curie temperature for Li, indicating a order-disorder phase transition, and a unimodal distribution for Nb, indicating a displacive phase transition.

SUPPLEMENTARY SECTION 6. FLUENCE

Supplementary Note 2

The fluence F [6] is defined as follows:

$$F = \frac{U}{A} = \frac{A}{A}\int_{-\infty}^{+\infty} dz\, u\,(z) \tag{S5}$$

where U is the electromagnetic energy of the pulse, A is the surface area of the sample, u is the volumetric energy density (constant over the surface), and z is the coordinate along the direction of propagation.

If we change the integration domain from space to time, by using $c = z/t$ where c is the speed of light in vacuum and $u = \varepsilon_0 \mathcal{E}^2\,(t)$, where ε_0 is the vacuum permittivity and $\mathcal{E}\,(t)$ is the electric field, we get

$$F = \varepsilon_0 c \mathcal{I} \qquad \text{with} \quad \mathcal{I} = \int_{-\infty}^{+\infty} dt\, \mathcal{E}^2\,(t) \tag{S6}$$

For the pulse adopted in this work, i.e. a plane wave of angular frequency ω with a gaussian envelope function of standard deviation σ, we can derive an explicit expression for $\mathcal{I}$:

$$\mathcal{I} = \int_{-\infty}^{+\infty} dt\, \mathcal{E}_{\max}^2 e^{-\frac{t^2}{\sigma^2}} \cos^2\,(\omega t) \tag{S7}$$

$$= \int_{-\infty}^{+\infty} dt\, \mathcal{E}_{\max}^2 e^{-\frac{t^2}{\sigma^2}} \left[\frac{1+\cos\,(2\omega t)}{2}\right] \tag{S8}$$

$$= \frac{\mathcal{E}_{\max}^2}{2}\left[\int_{-\infty}^{+\infty} dt\, e^{-\frac{t^2}{\sigma^2}} + \int_{-\infty}^{+\infty} dt\, e^{-\frac{t^2}{\sigma^2}} \cos\,(2\omega t)\right] \tag{S9}$$

$$= \frac{\mathcal{E}_{\max}^2}{2}\left[\sigma\sqrt{\pi} + \sigma\sqrt{\pi} e^{-\sigma^2\omega^2}\right] \tag{S10}$$

$$= \mathcal{E}_{\max}^2 \frac{\sigma\sqrt{\pi}}{2}\left(1 + e^{-\sigma^2\omega^2}\right) \tag{S11}$$

where we have used to property of the gaussian integral and the Fourier transform of a gaussian. This lead to an explicit relation between the fluence F and the parameters σ, ω and $\mathcal{E}_{\max}$ of the pulse:

$$F = \varepsilon_0 c\, \mathcal{E}_{\max}^2 \frac{\sigma\sqrt{\pi}}{2}\left(1 + e^{-\sigma^2\omega^2}\right) \tag{S12}$$

$\mathcal{E}$ [V/Å]	F [mJ/cm^2]
0.05	4.7
0.15	42.3
0.20	75.3
0.25	117.6
0.30	169.4

Supplementary Table I. Conversion between the electric field intensity shown in the main text and the fluence for a laser pulse with $\sigma = 80$ fs (equal to a FWHM of = 188 fs) and $\nu = 18$ THz.

SUPPLEMENTARY SECTION 7. PHONON DRIVING OF $LiNbO_3$

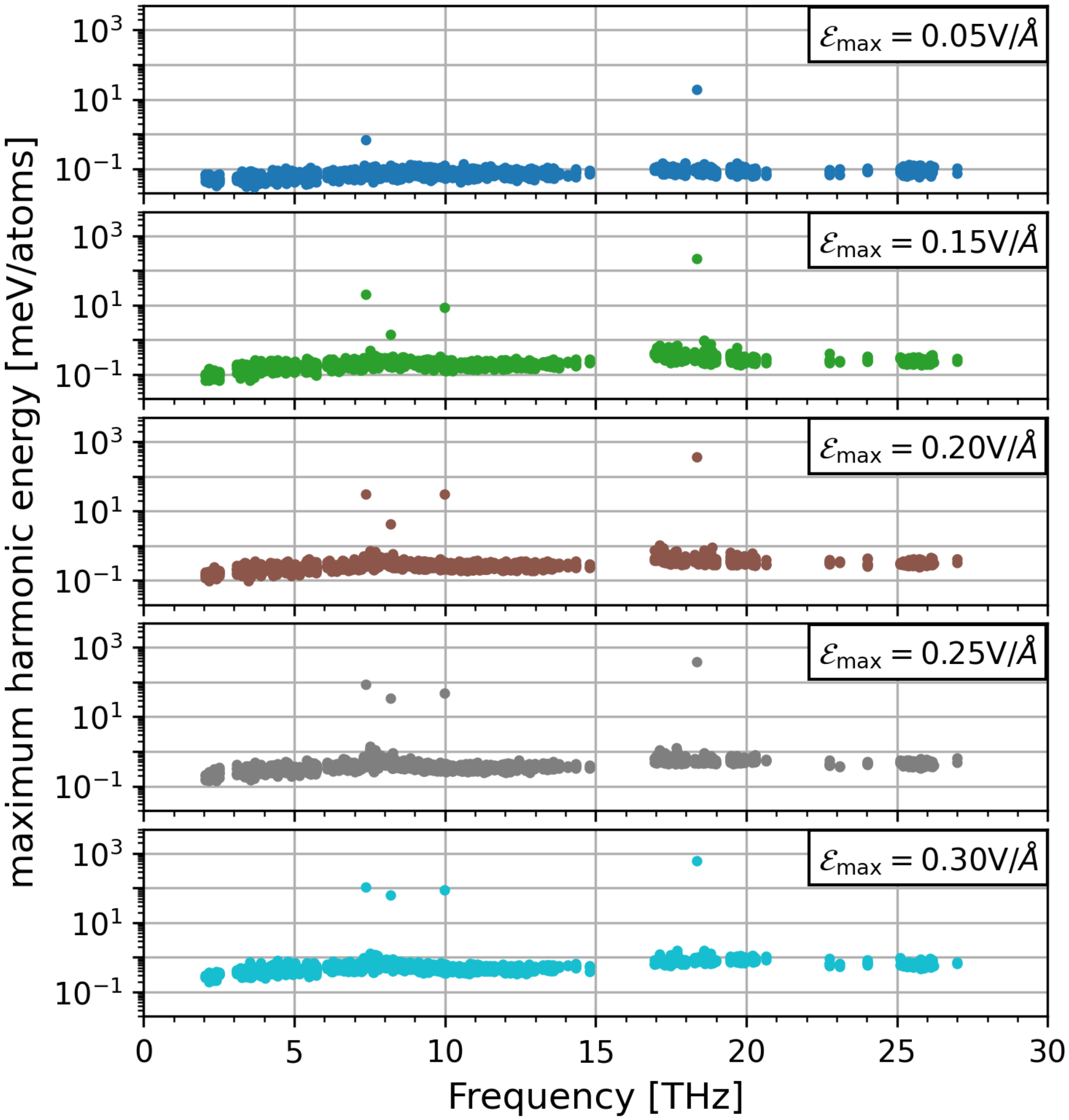

Supplementary Figure 6. Maximum harmonic energy assumed during the simulations by each the vibrational mode of the 4×4×4 $LiNbO_3$ supercell. Only 4 modes, identified to be Γ-point modes and shown in Figure 7 are excited (one directly by the pulse, and the other three by non-linear coupling). The projection procedure is described in SUPPLEMENTARY SECTION 8.

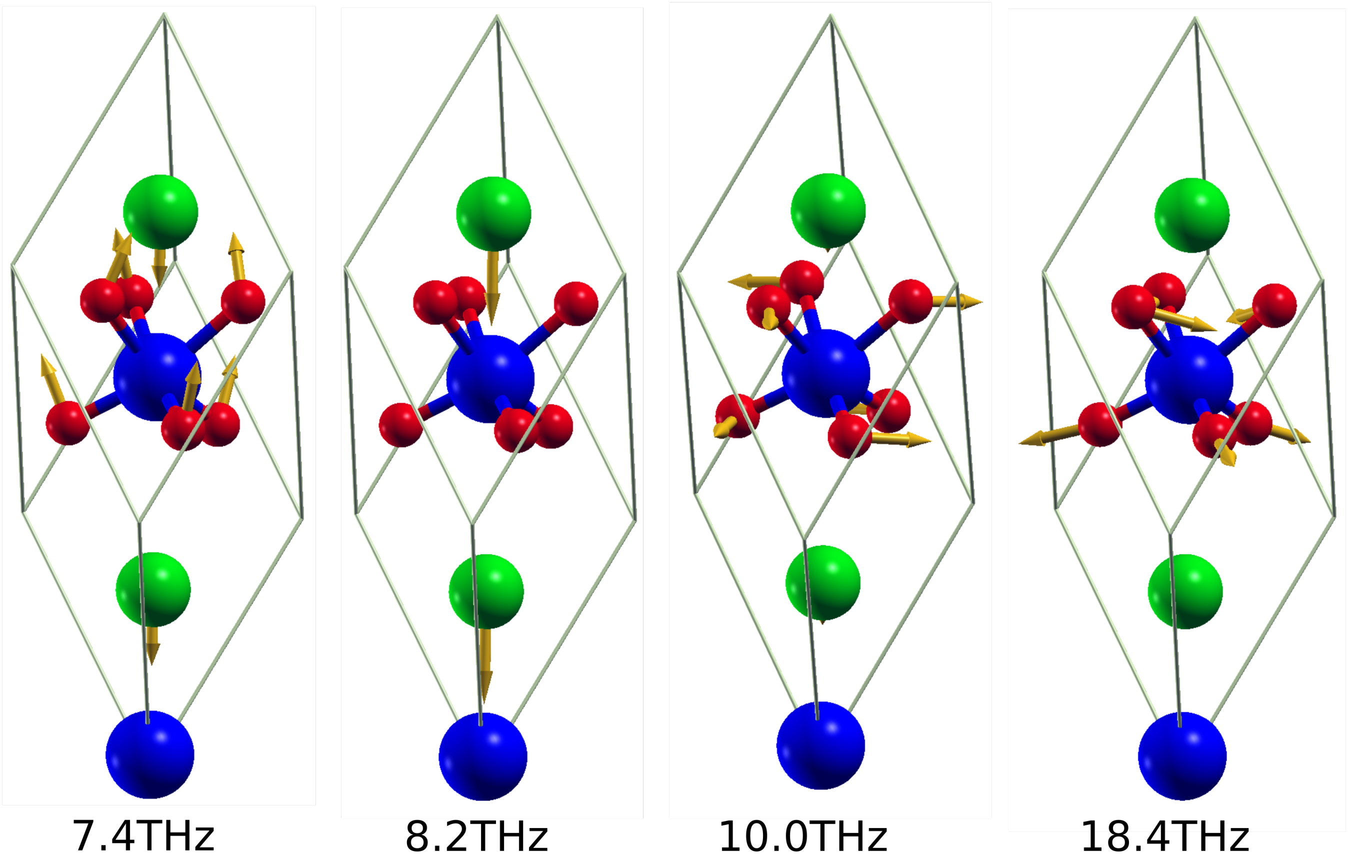

Supplementary Figure 7. The 4 infrared active A_1 phonon modes of $LiNbO_3$ excited during the laser pulse, with their respective frequencies. The pulse was tuned at 18 THz. The frequencies of these modes were computed with `phonopy` [7, 8]. The modes at 7.4 THz and 18.4 THz correspond to Q_{P} and Q_{IR}, respectively. The characters of the Q_{P} and Q_{IR} modes are in agreement with what reported in Ref. [9].

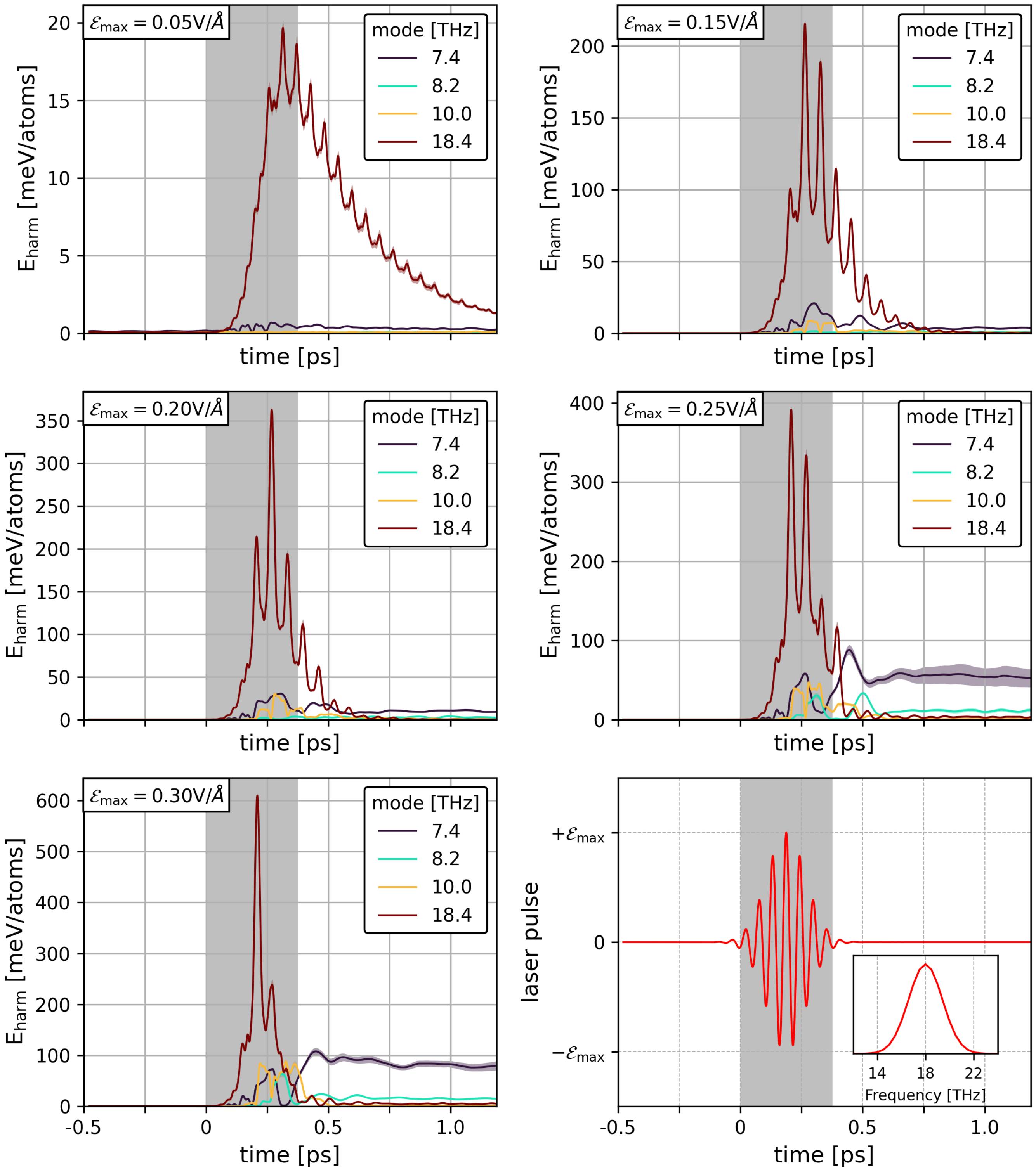

Supplementary Figure 8. Time evolution of the energy of the most excited modes due to the laser pulse. The pulse shape, described in the main text, is also shown in the last panel together with its power spectrum. The energies have been evaluated by projecting the trajectories along the harmonic normal modes, as exposed in SUPPLEMENTARY SECTION 8.

SUPPLEMENTARY SECTION 8. PROJECTION ONTO PHONON MODES

Supplementary Note 3

This section describes the procedure for projecting molecular dynamics trajectories onto the phonon modes of a system to obtain a mode-resolved picture of the dynamics. Using a compact notation, nuclear displacements $\boldsymbol{q}$ and velocities $\boldsymbol{v}$ are written as:

$$\boldsymbol{q} = (q_x^1, q_y^1, q_z^1, \ldots, q_x^{N_a}, q_y^{N_a}, q_z^{N_a}), \tag{S13}$$

$$\boldsymbol{v} = (v_x^1, v_y^1, v_z^1, \ldots, v_x^{N_a}, v_y^{N_a}, v_z^{N_a}). \tag{S14}$$

In the harmonic approximation, the potential energy U can be expressed as:

$$\mathcal{H} = \frac{1}{2}\boldsymbol{v}^{\mathrm{t}} \cdot \underline{\underline{M}} \cdot \boldsymbol{v} + \frac{1}{2}\boldsymbol{q}^{\mathrm{t}} \cdot \underline{\underline{\Phi}} \cdot \boldsymbol{q}, \tag{S15}$$

where $\underline{\underline{M}}$ is a diagonal mass matrix, and $\underline{\underline{\Phi}}$ is the force constants matrix. The related eigenvalue problem is:

$$\underline{\underline{D}} \cdot \underline{\underline{\boldsymbol{\varepsilon}}} = \underline{\underline{\boldsymbol{\varepsilon}}} \cdot \underline{\underline{\Lambda}}, \tag{S16}$$

where $\underline{\underline{D}} = \underline{\underline{M}}^{-1/2} \cdot \underline{\underline{\Phi}} \cdot \underline{\underline{M}}^{-1/2}$ is the dynamical matrix, and $\underline{\underline{\Lambda}}$ contains the eigenvalues ω_n^2, where ω_n are the angular frequencies of the normal modes. Displacements and velocities can be expressed in terms of normal modes:

$$\boldsymbol{q}(t) = \underline{\underline{M}}^{-1/2} \cdot \boldsymbol{\varepsilon} \cdot \tilde{\boldsymbol{q}}(t), \tag{S17}$$

$$\boldsymbol{v}(t) = \underline{\underline{M}}^{-1/2} \cdot \boldsymbol{\varepsilon} \cdot \underline{\underline{\Lambda}}^{1/2} \cdot \tilde{\boldsymbol{v}}(t), \tag{S18}$$

where $\tilde{\boldsymbol{q}}(t)$ and $\tilde{\boldsymbol{v}}(t)$ are the mode coefficients. These are computed from molecular dynamics trajectories by inverting the previous equations:

$$\tilde{\boldsymbol{q}}(t) = \underline{\underline{\boldsymbol{\varepsilon}}}^{\mathrm{t}} \cdot \underline{\underline{M}}^{1/2} \cdot \boldsymbol{q}(t), \tag{S19}$$

$$\tilde{\boldsymbol{v}}(t) = \underline{\underline{\Lambda}}^{-1/2} \cdot \underline{\underline{\boldsymbol{\varepsilon}}}^{\mathrm{t}} \cdot \underline{\underline{M}}^{1/2} \cdot \boldsymbol{v}(t). \tag{S20}$$

Modes with $\omega \approx 0$, corresponding to rigid translations and rotations, can be discarded for numerical stability. The harmonic energy E_n stored in each normal mode is given by:

$$E_n = \frac{1}{2}\omega_n^2 \left(\tilde{v}_n^2 + \tilde{q}_n^2\right) \tag{S21}$$

This provides a mode-resolved decomposition of the dynamics. The same reasoning can be extended to off-Γ modes, by considering the $\boldsymbol{k}$-dependence of all the previous quantities. A comprehensive exposition of the phonon theory can be found in Refs. [10, 11].

SUPPLEMENTARY SECTION 9. ACCURACIES OF THE TRAINED MODELS

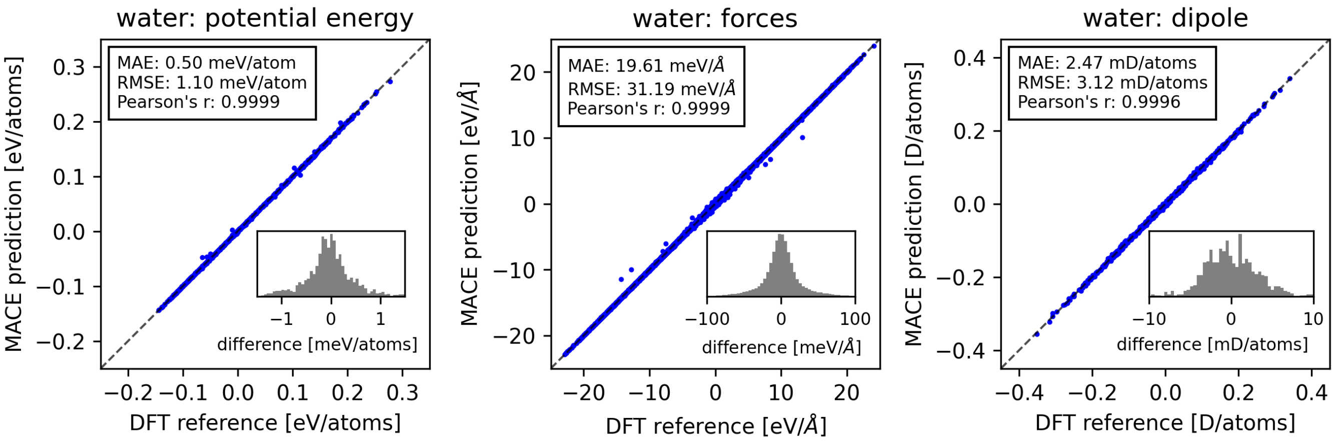

Supplementary Figure 9. Correlation plots of MACE predictions with DFT reference values for liquid water on the training dataset for potential energy, forces, and on the test dataset for the dipole. For forces and dipoles, all components are plotted together. Inset histograms show the distribution of prediction errors for these quantities. We note that the outlier points in the forces correlation plot correspond to a single structure in the dataset, in which dissociation of water molecules is present.

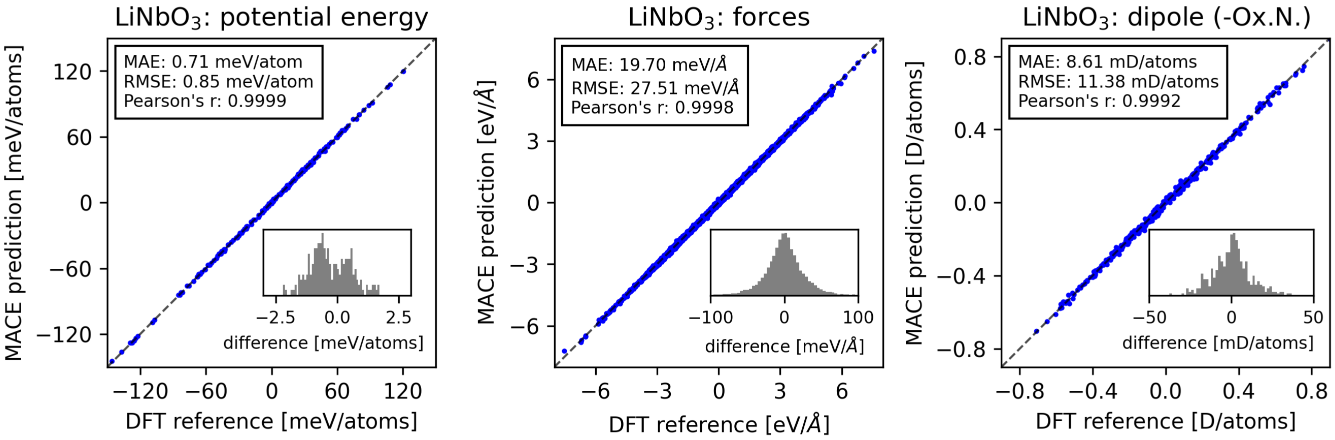

Supplementary Figure 10. Correlation plots of MACE predictions with DFT reference values for $LiNbO_3$ on the test dataset for potential energy, forces, and dipole. For forces and dipoles all components are plotted together. Inset histograms show the distribution of prediction errors for these quantities. In the plots for the dipole, the oxidation number contribution has been subtracted for easier visualization.

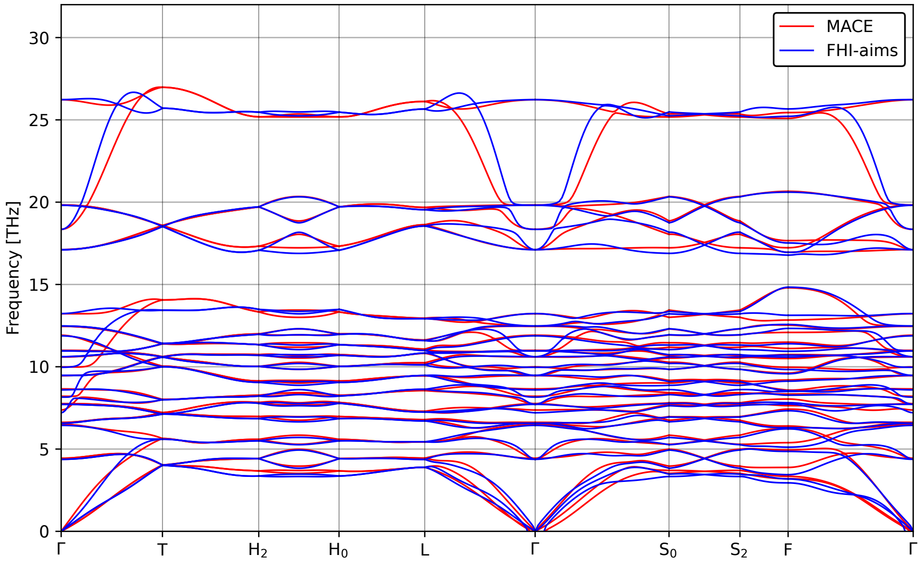

Supplementary Figure 11. Comparison of the phonon bands of $LiNbO_3$ (without non-analytical corrections) between the trained `MACE` potential and `FHI-aims`. The phonons have been computed using a 4×4×4 supercell and a 20×20×20 mesh using `phonopy` [7, 8]. The band paths along the Brillouin zone was generated using `seekpath` [12].

SUPPLEMENTARY SECTION 10. DIPOLE PREPROCESSING

Supplementary Note 4

The values of the dipoles $\boldsymbol{\mu}$ as computed from density-functional theory (DFT) need to be pre-processed in order to train any machine-learning (ML) model, since the values from different structures could lie on different branches.

In order to fix the *branch uncertainty*, we have followed the same procedure exposed in Ref. [13]: (i) construct a toy model for the dipole $\boldsymbol{\mu}^{\mathrm{ref}}$ that will be used as a reference value at varying nuclear coordinates $\boldsymbol{R}$; (ii) create a correlation plot of $\boldsymbol{\mu}^{\mathrm{DFT}}$ versus $\boldsymbol{\mu}^{\mathrm{ref}}$ for all the necessary structures; (iii) Shift the clusters of points along the bundle of lines $y = nx$ with $n \in \mathbb{Z}$, by the necessary number of quanta (along each $\boldsymbol{a}_\alpha$) such that all the point will lie along the same line. If the chosen model is sufficiently descriptive, all $\boldsymbol{\mu}^{\mathrm{DFT}}$ will belong to the same branch after this procedure.

There is a freedom of choice for the adopted toy model and for the chosen reference branch. We will explain it further in the following. Among the possible choices for a toy model we can cite: (i) partial charges model, (ii) linear model, (iii) a pre-trained ML model. A partial charges model is simply defined as $\boldsymbol{\mu}^{\mathrm{PC}}(\boldsymbol{R}) = \sum_I Q^I \boldsymbol{R}^I$ where Q^I are real-valued atomic charges. Such a model, adopted in Refs. [14], is well suited for molecular and liquid systems, such as bulk water, and it's fairly easy to implement, depending only on few parameters. A linear model, as the one implemented in Ref. [13], is defined as $\boldsymbol{\mu}^{\mathrm{LM}}(\boldsymbol{R}) = \boldsymbol{Z}^*_{\mathrm{ref}} \cdot (\boldsymbol{R} - \boldsymbol{R}_{\mathrm{ref}})$, and it's suitable for solid systems where it's easy to identify a reference structure $\boldsymbol{R}_{\mathrm{ref}}$ (usually a paraelectric ground state). Such a model is effective for solids because all the considered structures (at least for low enough temperature) can be assumed close enough to $\boldsymbol{R}_{\mathrm{ref}}$, not showing any pronounced non-linearities. However, this approach depends on a number of parameters which are linear in the system size and cannot be transferred "out-of-the-box" to cells with a different number of atoms, since Z^*_{ref} and $\boldsymbol{R}_{\mathrm{ref}}$ are "cell-dependent". A pre-trained ML model for the dipole has the advantage to grasp nonlinearities in the dipole, but of course it can be used only at a second stage while at a first stage one of the previous model has to be adopted. However, such a model allows to generate a self-consistent workflow able to correct data that were placed in the wrong branch on a previous stage.

The correction of the data by using one of the previously mentioned method will move all the data to the same branch, which is determined by the chosen reference model. However, as already recognized in Ref. [13], even though from the physical point of view any branch is equivalent, from a practical point of view this is not the case when working with $E(3)$-equivariant models. In a $E(3)$-equivariant framework, the dipole $\boldsymbol{\mu}$ is odd with respect to nuclear coordinate $\boldsymbol{R}$, i.e. $\boldsymbol{\mu}(-\boldsymbol{R}) = -\boldsymbol{\mu}(\boldsymbol{R})$ (where a rotation of the lattice vector $\boldsymbol{a}_\alpha$ is understood as well). This means that we can not shift the values of the dipoles by any $e\boldsymbol{a} \cdot \boldsymbol{n}$ since:

$$\begin{aligned} \boldsymbol{\mu}(\boldsymbol{R}) \longrightarrow \boldsymbol{\mu}'(\boldsymbol{R}) &= \boldsymbol{\mu}(\boldsymbol{R}) + e\boldsymbol{a} \cdot \boldsymbol{n} \\ \boldsymbol{\mu}(-\boldsymbol{R}) \longrightarrow \boldsymbol{\mu}'(-\boldsymbol{R}) &= \boldsymbol{\mu}(-\boldsymbol{R}) + e\boldsymbol{a} \cdot \boldsymbol{n} = -\boldsymbol{\mu}(\boldsymbol{R}) + e\boldsymbol{a} \cdot \boldsymbol{n} \\ \boldsymbol{\mu}'(\boldsymbol{R}) &\neq -\boldsymbol{\mu}'(-\boldsymbol{R}) \end{aligned} \tag{S22}$$

This means that the use of a $E(3)$-equivariant ML models *imposes* to chose the only branch where equivariance can be satisfied. A possible way to achieve this goal is to use a reference model which is explicitly $E(3)$-equivariant, such as a point-charges model (being linear in $\boldsymbol{R}$). In this paper we have used at a first stage a point charges model where the partial-charges Q were set equal to the oxidation numbers $\mathcal{N}_I$ for $LiNbO_3$ and half of their value for bulk water, and on a second stage the trained ML model in a self-consistent manner in order to ensure that all the data were placed in the correct branch.

SUPPLEMENTARY REFERENCES

[1] D. McQuarrie, *Statistical Mechanics* (University Science Books, 2000).

[2] B. J. Braams, T. F. Miller, and D. E. Manolopoulos, Sum rule constraints on kubo-transformed correlation functions, Chemical Physics Letters **418**, 179 (2006).

[3] M. Rossi, M. Ceriotti, and D. E. Manolopoulos, How to remove the spurious resonances from ring polymer molecular dynamics, The Journal of Chemical Physics **140**, 234116 (2014).

[4] M. Ceriotti, M. Parrinello, T. E. Markland, and D. E. Manolopoulos, Efficient stochastic thermostatting of path integral molecular dynamics, The Journal of Chemical Physics **133**, 124104 (2010), https://pubs.aip.org/aip/jcp/article-pdf/doi/10.1063/1.3489925/13343002/124104_1_online.pdf.

[5] F. Bernhardt, L. M. Verhoff, N. A. Schäfer, A. Kapp, C. Fink, W. A. Nachwati, U. Bashir, D. Klimm, F. E. Azzouzi, U. Yakhnevych, Y. Suhak, H. Schmidt, K.-D. Becker, S. Ganschow, H. Fritze, and S. Sanna, Ferroelectric to paraelectric structural transition in $LiTaO_3$ and $LiNbO_3$, Phys. Rev. Mater. **8**, 054406 (2024).

[6] R. Paschotta, Fluence, RP Photonics Encyclopedia.

[7] A. Togo, L. Chaput, T. Tadano, and I. Tanaka, Implementation strategies in phonopy and phono3py, J. Phys. Condens. Matter **35**, 353001 (2023).

[8] A. Togo, First-principles phonon calculations with phonopy and phono3py, J. Phys. Soc. Jpn. **92**, 012001 (2023).

[9] R. Mankowsky, A. von Hoegen, M. Först, and A. Cavalleri, Ultrafast Reversal of the Ferroelectric Polarization, Phys. Rev. Lett. **118**, 197601 (2017).

[10] A. Rigamonti and P. Carretta, *Structure of matter* (Springer, 2007).

[11] N. W. Ashcroft, N. D. Mermin, and S. Rodriguez, Solid state physics, American Journal of Physics **46**, 116 (1978).

[12] Y. Hinuma, G. Pizzi, Y. Kumagai, F. Oba, and I. Tanaka, Band structure diagram paths based on crystallography, Computational Materials Science **128**, 140 (2017).

[13] L. Gigli, M. Veit, M. Kotiuga, G. Pizzi, N. Marzari, and M. Ceriotti, Thermodynamics and dielectric response of $BaTiO_3$ by data-driven modeling, npj Computational Materials **8**, 209 (2022).

[14] A. Jana, S. Shepherd, Y. Litman, and D. M. Wilkins, Learning electronic polarizations in aqueous systems, Journal of Chemical Information and Modeling 10.1021/acs.jcim.4c00421 (2024).
